# Loss of α1,6-fucosyltransferase suppressed liver regeneration: implication of core fucose in the regulation of growth factor receptor-mediated cellular signaling

**DOI:** 10.1038/srep08264

**Published:** 2015-02-05

**Authors:** Yuqin Wang, Tomohiko Fukuda, Tomoya Isaji, Jishun Lu, Wei Gu, Ho-hsun Lee, Yasuhito Ohkubo, Yoshihiro Kamada, Naoyuki Taniguchi, Eiji Miyoshi, Jianguo Gu

**Affiliations:** 1 Division of Regulatory Glycobiology, Tohoku Pharmaceutical University, Sendai, Miyagi, 981-8558, Japan; 2 Department of Radiopharmacy, Tohoku Pharmaceutical University, Sendai, Miyagi, 981-8558, Japan; 3 Department of Molecular Biochemistry and Clinical Investigation, Osaka University Graduate School of Medicine, Osaka, 565-0871, Japan; 4 Disease Glycomics Team, RIKEN, Wako, Saitama, 351-0198, Japan

## Abstract

Core fucosylation is an important post-translational modification, which is catalyzed by α1,6-fucosyltransferase (Fut8). Increased expression of Fut8 has been shown in diverse carcinomas including hepatocarcinoma. In this study, we investigated the role of Fut8 expression in liver regeneration by using the 70% partial hepatectomy (PH) model, and found that Fut8 is also critical for the regeneration of liver. Interestingly, we show that the Fut8 activities were significantly increased in the beginning of PH (~4d), but returned to the basal level in the late stage of PH. Lacking Fut8 led to delayed liver recovery in mice. This retardation mainly resulted from suppressed hepatocyte proliferation, as supported not only by a decreased phosphorylation level of epidermal growth factor (EGF) receptor and hepatocyte growth factor (HGF) receptor in the liver of Fut8^−/−^ mice *in vivo*, but by the reduced response to exogenous EGF and HGF of the primary hepatocytes isolated from the Fut8^−/−^ mice. Furthermore, an administration of L-fucose, which can increase GDP-fucose synthesis through a salvage pathway, significantly rescued the delayed liver regeneration of Fut8^+/−^ mice. Overall, our study provides the first direct evidence for the involvement of Fut8 in liver regeneration.

The adult liver has a remarkable capacity to regenerate, which makes it possible to use partial livers from living donors for transplantation. However, certain hepatic conditions, including cirrhosis, steatosis, and conditions due to old age, also have impaired liver regeneration that results in increased morbidity and mortality in response to liver transplantation^1^. Therefore, in the past decade, numerous studies have been focused on dissecting the molecular mechanisms underlying liver regeneration.

Seventy percent partial hepatectomy (PH) is the most common technique that is used to study the regeneration of liver. Namely, it describes a surgical procedure which removes 70% of liver mass in rodents (rats and mice). Due to the multi-lobed structure of the rodent liver, three of the five liver lobes (representing 70% of its liver mass) can be removed. The residual lobes enlarge and reconstitute the original size of the liver within 2 weeks^2^,^3^. Regeneration after PH is a complicated process. At the cellular level, it proceeds with the coordinated proliferation of all types of mature hepatic cells. Among these, it has been generally accepted that the restoration of liver volume depends mainly on the proliferation of hepatocytes^4^. This is not only because hepatocytes account for about 80% of liver weight and 70% of all liver cells, but also they are the first cells to enter into DNA synthesis and produce mitogenic signals for other hepatic cells^4^,^5^. Molecularly, PH triggers multiple intracellular signaling cascades (RAS/mitogen-activated protein kinase (MAPK) signaling, c-Met signaling, etc), leading to great changes in the expression of genes associated with cell proliferation^1^,^6^. The convergence of these signaling pathways has been reportedly mediated via epidermal growth factor receptor (EGFR) and hepatocyte growth factor receptor (HGFR, also called c-Met)^4^. Blocking the EGFR- or c-Met-mediated signaling pathway could cause a severe delay of liver regeneration. In addition to the expression level of EGFR and c-Met proteins, it has been shown that the post-translational modification of these receptors such as ubiquitination, phosphorylation, and glycosylation also plays a crucial role in the regulation of these signaling pathways^7^,^8^.

Fucosylation is one type of glycosylation. It describes the attachment of a fucose residue to *N*-glycans, *O*-glycans, and glycolipid catalyzed by a family of enzymes called fucosyltransferases (Futs)^9^. Among these, α1,6-fucosyltransferase (Fut8) is the only enzyme that catalyzes the transfer of a fucose from GDP-fucose to the innermost GlcNAc residue via α1,6-linkage to form core fucosylation in mammals as shown in Figure 1c. The enzymatic products, core fucosylated *N*-glycans, are widely distributed in a variety of glycoproteins and have been shown to play important roles in cell signaling. As examples, we previously showed that core fucosylation is crucial for the ligand binding affinity of TGF-β1 receptor^10^, EGF receptor^11^, and integrin α3β1^12^. Lacking the core fucose of these receptors led to a marked reduction in their ligand-binding ability and downstream signaling. Recently, our group found that a loss of core fucose on activin receptors resulted in an enhancement of the formation of activin receptor complexes, which constitutively activated intracellular signaling^13^. These studies indicate that core fucosylation is able to negatively or positively affect signaling pathways through regulation of receptor binding ability.

Abnormal expression of Fut8 has been pathologically correlated with diverse carcinomas including liver^14^, ovarian^15^, lung^16^ and colorectal cancers^17^. Recently it was reported that core fucosylation on some glycoproteins, such as vitronectin, increased during liver regeneration after PH^18^. However, the underlying mechanisms remain poorly understood. Here, we investigated the role of Fut8 in liver regeneration and showed for the first time that core fucosylation is physiologically associated with the liver regeneration. In particular, we show that the liver regeneration was significantly inhibited in Fut8 deficient (Fut8^−/−^) and Fut8 hetero (Fut8^+/−^) mice as compared to wild type (Fut8^+/+^) mice. It is intriguing that this effect could be attenuated by L-fucose supplementation in the Fut8^+/−^ mice. Moreover, intracellular signaling analysis using primary hepatocytes isolated from Fut8^+/+^ and Fut8^−/−^ mice clearly demonstrated that Fut8 is important for the initiation of hepatocyte proliferation. Taken together, our data here provide novel insight for the function of core fucosylation in liver regeneration.

## Results

### 70% PH induced the expression of Fut8

**Figure 1:**
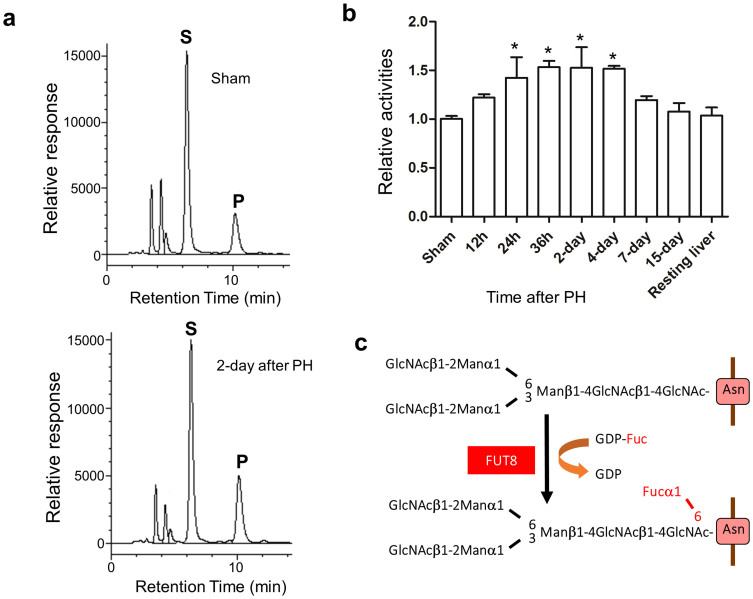
The activities of Fut8 were increased after 70% partial hepatectomy (PH). The liver tissues were harvested for the determination of enzyme activities at indicated times as described in “Methods”. (a) A representative elution pattern on HPLC for Fut8 activities in Fut8^+/+^ mouse with (left panel) or without (right panel) PH. S: substrate; P: product. (b) The quantitative assay for enzyme activities in Fut8^+/+^ mice after PH. *, *P* < 0.05, compared to the group without PH (sham), which was set as 1, n = 3. (c) Reaction for synthesis of α1,6-fucose.

It has been reported that lacking N-acetylglucosaminyltransferase III suppressed the liver tumor progression and liver regeneration in mice, indicating the importance of glycosylation in liver^19^. In the present study, we investigated the roles of Fut8 in liver regeneration. Firstly, we chose to use HPLC to examine the enzyme activities of Fut8 by in the liver tissues at different time points after 70% PH, since the expression level of Fut8 in liver is much lower than that in other tissues under physiological conditions, and it is difficult to detect endogenous Fut8 by anti-Fut8 antibody even after the induction by PH. As shown in Figure 1a and 1b, the Fut8 activities were increased in the first 4 days after operation, and returned to normal levels after liver mass is restored. The similar pattern was also observed in mRNA expression confirmed by RT-PCR (data not shown). On the other hand, the expression levels of L-fucosidase after PH were not changed confirmed by RT-PCR (data not shown). These data indicated that the induction of Fut8 expression might be required for liver regeneration.

### Loss of Fut8 inhibited recovery of liver mass after a two-third liver resection

**Figure 2:**
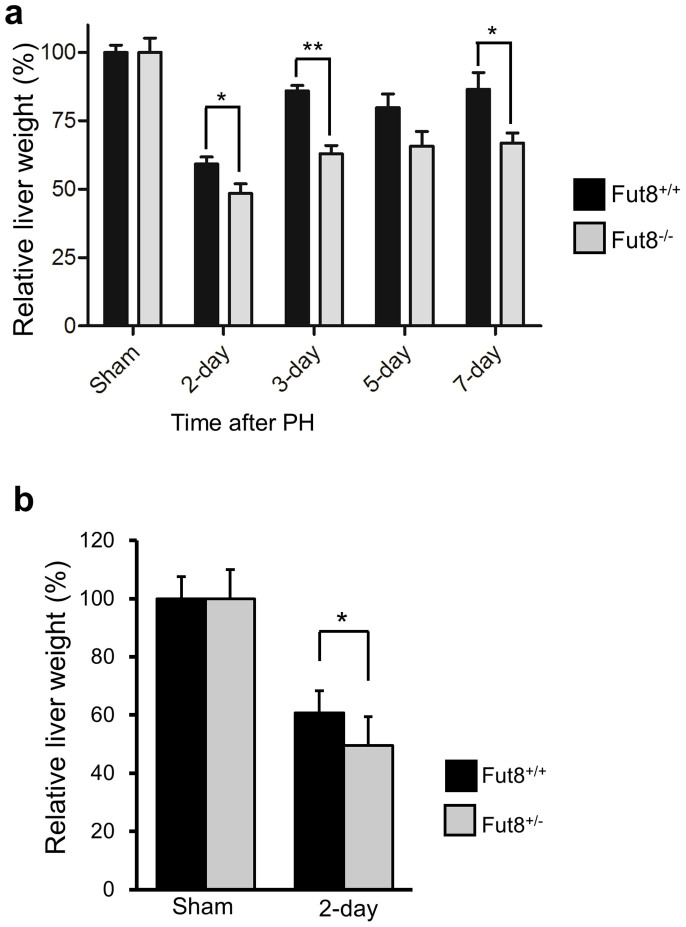
Fut8 expression was required for liver regeneration after PH. 7- to 8-week-old mice were surgically resected as described in “Methods”, and then the livers were harvested at the indicated times. (a) Relative liver weight (liver vs whole body) at the indicated times after 70% PH. The sham group was set as 100%. Each set of the reported data was obtained from at least 5 individuals of Fut8^+/+^ and Fut8^−/−^ mice. *, *P* < 0.05; **, *P* < 0.01. (b) Comparison of relative weight at 2 days after PH between Fut8^+/+^ and Fut8^+/−^ mice (C57BL/6 genetic background). Each data was obtained from at least 8 individuals. *, *P* < 0.05, compared with the Fut8^+/+^ mice.

To testify the hypothesis above, we performed a 70% PH on both Fut8^+/+^ and Fut8^−/−^ mice, and analyzed the restoration of their livers. Interestingly, the regeneration index calculated as an increase in liver-to-body weight ratio was significantly lower in Fut8^−/−^ mice than that in Fut8^+/+^ mice (Figure 2a). Furthermore, a decrease in liver regeneration was also observed in the Fut8^+/−^ mice during the first 2 days (Figure 2b). The results above indicated that the liver regeneration was inhibited in Fut8^−/−^ mice as compared to Fut8^+/+^ mice.

**Figure 3:**
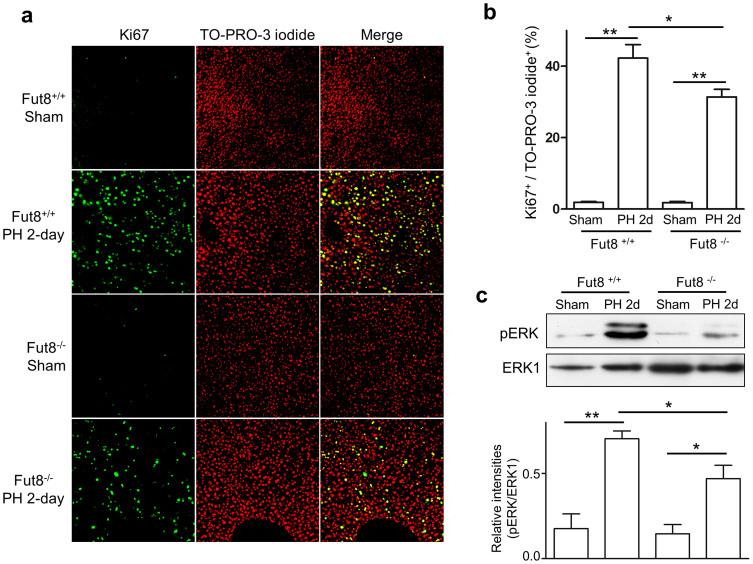
Cell proliferation was suppressed in the livers of Fut8^−/−^ mice. (a) Immunostaining for liver tissues (10 μm frozen section) of Fut8^+/+^ and Fut8^−/−^ mice using anti-Ki67 antibody (200 × field). The positive cells of the immunostaining were labeled with the green spots (left panel), and the nuclei were labeled by TO-PRO-3 iodide (red spots, middle panel). (b) The quantitative data were obtained from at least 3 mice in each group. **, *P* < 0.01. (c) Equal protein of liver lysates at day 2 after PH were separated by 10% SDS-PAGE and blotted with anti-phospho-ERK and anti-ERK1 antibodies. The quantitative data were obtained from 3 mice in each group. *, *P* < 0.05, **, *P* < 0.01.

Liver regeneration was achieved by the coordinated proliferation of all types of mature hepatic cells^2^. Consistent with the results above, quantitative assessment of Ki67 by immunostaining revealed little difference between Fut8^−/−^ and Fut8^+/+^ mice without PH, while, the percentage of Ki67 positive versus TO-PRO-3 iodide positive cells in the livers of Fut8^−/−^ mice were markedly less than that in Fut8^+/+^ mice at day 2 after PH (Figure 3a and 3b). These differences in cell proliferation were further reflected by the cell proliferation signaling. As shown in figure 3c, the phosphorylation levels of ERK were remarkably lower in the Fut8^−/−^ mice as compared with Fut8^+/+^ mice, although the MAPK signaling pathways were activated by PH in both Fut8^+/+^ and Fut8^−/−^ mice. Overall, these data indicated that the delayed liver recovery in Fut8^−/−^ mice resulted from the lower cell proliferation.

### L-fucose administration in Fut8^+/−^ mice attenuated the inhibitory effect in cell proliferation as described above

**Figure 4:**
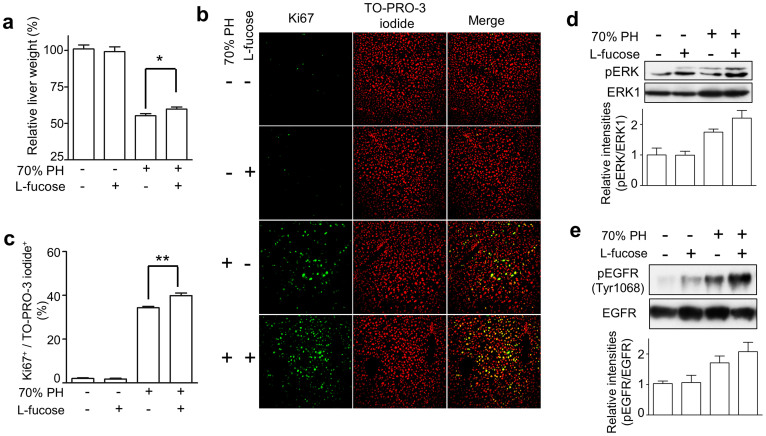
L-fucose supplementation attenuated the decreased regeneration of Fut8^+/−^ mice. (a) Relative liver weight (liver vs whole body) at 2-day after PH in Fut8^+/−^ mice with or without administration of L-fucose. Prior to operation, 6-week old Fut8^+/−^ mice were administrated with L-fucose at 4 g/L in water for 12 days, and then the livers were harvested at 48 hours after PH. The sham group without L-fucose treatment was set as 100%. *, *P* < 0.05, compared with the mice without L-fucose treatment (n > 10 mice). (b) Immunostaining for liver tissues using anti-Ki67 antibody (200 × field). (c) The quantitative data were obtained from at least 3 mice in each group, *, *P* < 0.05. Equal protein of liver lysates at day 2 after PH were separated by SDS-PAGE (10% for pERK/ERK1, 7% for pEGFR/EGFR) and blotted with anti-phospho-ERK and anti-ERK1 antibodies. The quantitative data were obtained from 3 mice in each group.

GDP-fucose is the donor for fucosyltransferases. It is known that two pathways for the synthesis of GDP-fucose in mammalian cells, the GDP-mannose-dependent *de novo* pathway and the free fucose-dependent salvage pathway^20^. And what is more, administration of oral L-fucose, an enhancement of the salvage pathway, has been proven useful for correction of fucosylation defects in leukocyte adhesion deficiency type II (LAD II) patients^21^. To determine whether enhancing GDP-fucose salvage pathway could complement the delayed liver regeneration of the Fut8^+/−^ mice as described above, we checked the effects of L-fucose supplementation in the Fut8^+/−^ mice. Interestingly, an oral administration of L-fucose significantly accelerated liver regeneration of the Fut8^+/−^ mice, but did not affect sham mice (Figure 4a). Consistently, in contrast to the little difference in the case of livers without 70% PH, immunostaining with Ki67 showed the ratio of Ki67^+^ to TO-PRO-3 iodide^+^ cells in the livers treated by PH were clearly increased after L-fucose administration (Figure 4b and 4c). Moreover, as shown in figure 4d and 4e, the phosphorylation levels of ERK and EGFR were induced in Fut8^+/−^ mice after PH. Furthermore, the L-fucose administration up-regulated their phosphorylation levels, although there was no significant difference between the mice treated with or without L-fucose by statistical analysis. These results further suggest that Fut8 and its products are important for cell proliferation in liver regeneration.

### The intracellular signaling was inhibited in the Fut8^−/−^ primary hepatocytes upon stimulation with EGF or HGF

**Figure 5:**
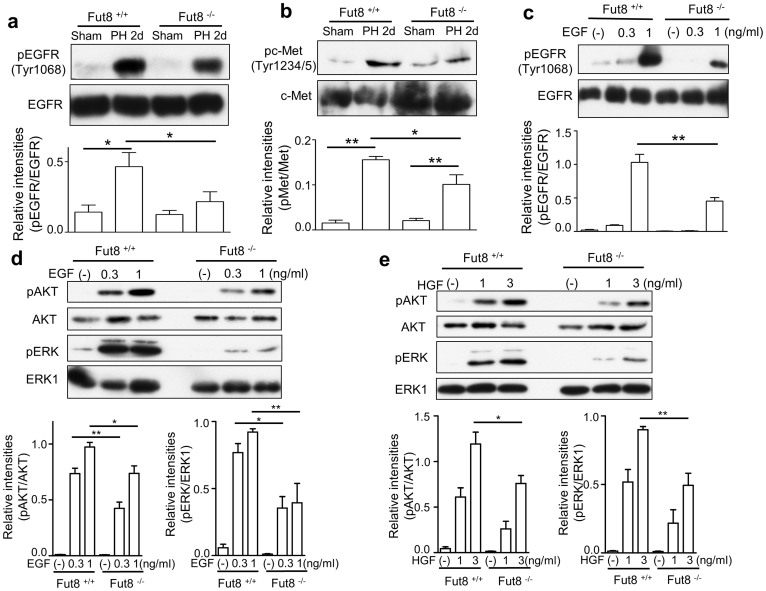
Intracellular signaling was suppressed in Fut8^−/−^ mice upon either PH or EGF and HGF stimulation. 8-week-old Fut8^+/+^ or Fut8^−/−^ mice were surgically resected as described in “Methods”, and then the livers were harvested at 2 days. The liver homogenates were separated by 7% SDS-PAGE and blotted with anti-EGFR and anti-phospho-EGFR antibodies (a), and anti-c-Met and anti-phospho-c-Met antibodies (b). The quantitative data were obtained from 3 mice in each group. *, *P* < 0.05, **, *P* < 0.01. The primary hepatocytes isolated from 8-week old Fut8^+/+^ and Fut8^−/−^ mice were cultured in DMEM containing with 10% FBS for 12 h, and then cultured under DMEM containing with 0.1% FBS for 24 hours. After the starvation, these cells were stimulated with or without EGF at indicated concentrations for 5 min (c and d), or HGF at indicated concentrations for 10 min (e). The cell lysates were immunoblotted with anti-pEGFR and anti-EGFR, anti-pAKT and anti-AKT antibodies, anti-pERK and anti-ERK1 antibodies. The quantitative data were obtained from at least 3 independent experiments, *, *P* < 0.05, **, *P* < 0.0 1.

The EGF and HGF are major mitogens for hepatocytes in the regenerating liver. Lacking EGFR or c-Met in mice resulted in the liver regeneration abnormalities^22^,^23^. To determine whether the delayed liver recovery in the Fut8^−/−^ mice is due to the impaired EGFR and/or c-Met signaling, we tested the expression levels of the key effectors in these signaling pathways. As shown in Figure 5a and b, although c-Met and EGFR associated signaling pathways were activated in both Fut8^+/+^ and Fut8^−/−^ mice 2 days post PH, the levels of phosphorylated c-Met (Tyr1234/5) and EGFR (Tyr1068) in Fut8^−/−^ mice were obviously lower than that in Fut8^+/+^ mice. These results indicated that loss of Fut8 impaired EGFR and c-Met associated signaling during liver regeneration.

To further corroborate the results above *in vitro*, we examined the downstream signaling cascades of EGF or HGF using the primary hepatocytes isolated from Fut8^+/+^ and Fut8^−/−^ mice. Consistently, the treatments with EGF or HGF significantly increased the expression levels of phosphorylated ERK and AKT in the Fut8^+/+^ cells. However, these increases were greatly suppressed in the Fut8^−/−^ cells (Figure 5d and e). The results above clearly demonstrated that the impaired regeneration in Fut8^−/−^ livers was due, at least mainly, to the down-regulated EGFR- and c-Met-mediated signalings in hepatocytes.

## Discussion

**Figure 6:**
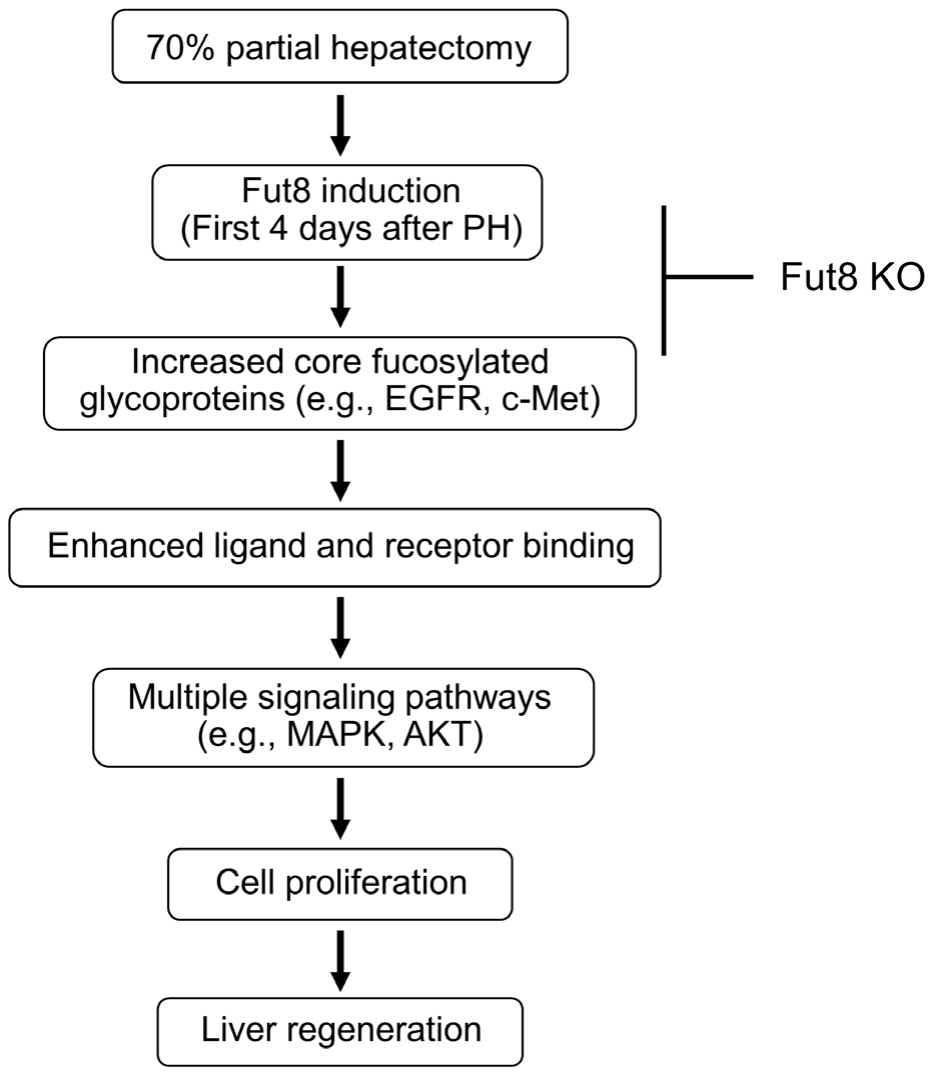
Proposed molecular mechanisms for the delayed liver regeneration in Fut8^−/−^ mice. It is well known that the 70% partial hepatectomy could activate several cell proliferation associated signaling pathways including Ras/MAPK signaling, c-Met signaling, and Akt/mammalian targets of rapamycin (mTOR) signaling, which up-regulate the cell proliferation, and consequently lead to the restoration of liver. Loss of core fucosylation on growth factor receptors such as EGFR and c-Met may alter their conformation and impair their ligand binding, thereby inhibiting their downstream signalings, and ultimately suppressing the cell proliferation. Overall, a loss of Fut8 gene results in a decrease in liver regeneration.

In the present study, we used a well-established regeneration model, to investigate the functions of Fut8 in liver regeneration, and found the following: i) The expression of Fut8 was markedly up-regulated during the regenerating process in the Fut8^+/+^ mice; ii) the liver regeneration was greatly inhibited in Fut8^−/−^ mice compared to Fut8^+/+^ mice; iii) L-fucose supplementation could reverse the delayed regeneration in Fut8^+/−^ mice; and, iv) the responses to growth factors such as EGF and HGF, were decreased in Fut8 deficient hepatocytes compared to wild-type hepatocytes. Overall, this study marks the first clear demonstration of the biological functions of Fut8 in the liver, suggesting that core fucosylation plays important roles in liver regenerating progression as shown in Fig. 6.

Liver regeneration after PH is a complicated process with the coordinated proliferation of all types of mature hepatic cells, which involves numerous molecules and signaling pathways^1^,^2^,^6^,^24^. Among these, the EGFR-mediated signaling has been reported to be critical for liver regeneration^24^. Lacking EGFR in hepatocytes increased the mouse mortality rate after PH, and delayed the hepatocyte proliferation^23^, although little effect was observed on liver function. We have previously shown that core fucosylation on EGFR is required for its binding to EGF and downstream signaling in embryonic fibroblast cells^11^. Therefore, it is reasonable to consider that the delayed liver recovery of Fut8^−/−^ mice could be attributed, at least mainly, to the loss of the core fucosylation on the EGFR protein (Figure 5c and d). In agreement with this hypothesis, we found here that knockout of Fut8 led to an inhibition of the EGFR-mediated signaling cascade both *in vivo* and *in vitro*.

In addition to EGFR, c-Met has also been shown to play an irreplaceable role in liver regeneration. c-Met gene deficient or suppressed by shRNAs significantly inhibited the proliferation of hepatocytes after PH^22^,^25^,^26^. In the present study, we found that knockout of Fut8 also attenuated the response to an HGF stimulus in primary hepatocytes (Figure 5e). Since c-Met is also a core fucosylated protein which had been confirmed by using human cell lines (data not shown), one possibility for this attenuated response is that like EGFR, the core fucosylation on c-Met may be necessary for its ligand binding and downstream signaling as well. Obviously, we could not exclude other possibilities. Recently, Tobias Speicher et al. reported that the β1-integrin knockout or knockdown in mice inhibited liver regeneration by impairing the ligand-induced phosphorylation of EGFR and c-Met, as well as their downstream signalings^27^. Considering also that α3β1 integrins were highly modified by Fut8 and loss of core fucosylation could result in the malfunction of β1-integrin^12^, Fut8 may also affect the c-Met-mediated signaling in the liver regeneration by regulating the core fucosylation status of β1-integrin. Further investigation is required to confirm the hypotheses above.

Increasing evidence indicated the importance of core fucosylation in protein-protein interaction, and we proposed here that Fut8 may affect the liver regeneration through modulating some associated receptor-ligand bindings. However, the mechanistic roles of Fut8 underlying the protein-protein interaction remain poorly understood. Recently, two research teams determined the complex structures of glycosylated FcγRIIIa and human core fucosylated or afucosylated Fc of IgG^28^,^29^. Interestingly, the crystal structures indicated that core fucose depletion increased the incidence of the active conformation of the Tyr-296 of Fc, and thereby accelerated the formation of the high-affinity complex with its receptor. These findings clearly explained why the lack of a core fucose on IgG could greatly enhance antibody-dependent cell-mediated cytotoxicity as previously reported^30^,^31^. From a more general viewpoint, these studies provide direct evidence for the mechanistic roles of Fut8 in different biological processes, where the attachment of core fucose leads to an alteration of glycoprotein conformation, which determines its protein dynamics coupled with the selection of protein-protein interactions and complex formation, and consequently affects the intracellular signaling pathways.

The excellent results of liver transplantation have led to an increasing number of patients on the waiting list, while the number of liver donors remains stable^1^,^32^. Studies on potential hepatoprotective factors in liver injury may contribute to increasing the success ratio of liver transplantation. Here, we showed that liver regeneration is significantly inhibited in Fut8^−/−^ mice. Moreover, L-fucose administration could partially complement the delayed liver recover in Fut8^+/−^ mice. However, it had no effect on the liver growth of the Fut8^+/−^ sham mice, so we hypothesize that Fut8 exerts its regulatory functions in the liver only after some stimulus such as 70% PH. Clearly, it needs to confirm this idea and elucidate the underlying mechanisms in future. Nevertheless, the current study provides clear evidence for the effect of L-fucose supplementation on liver regeneration in mice and indicates the important role of Fut8 in liver regeneration.

## Methods

### Mice

The Fut8-deficient mice line used for these studies has been described previously^10^,^33^. Male mice on an ICR background at 6 to 8 weeks of age were used for the experiments in the present study, comparing Fut8^−/−^ animals with Fut8^+/+^ littermates. Mice were housed in a temperature-controlled room with a 12-h dark/12-h light cycle. Food and water were provided *ad libitum*. The present study was approved by the Institutional Animal Care and Use Committee of Tohoku Pharmaceutical University, Japan.

### 70% partial hepatectomy

All experiments were carried out in accordance with relevant guidelines and regulations. For liver regeneration studies, 7- to 8- week-old mice were anesthetized with pentobarbital sodium and subjected to mid-ventral laparotomy with a two-third liver resection, as previously described^34^,^35^. The left and median liver lobes were surgically resected without injuring the remaining liver tissue. The removed parts represented the resting liver. At least three mice from each group were euthanized at each analysis time point. For L-fucose (Nacalai tesque Inc.) supplementation, 6-week-Fut8^+/-^ mice were orally administrated with L-fucose (4 g/L in water) for 12 days prior to partial hepatectomy (PH), and then the livers were harvested at 48 h after operation.

### Immunostainings

The hepatic lobules were assessed based on 10 μm frozen sections. Proliferative cells in the liver were detected through immunostaining with a monoclonal antibody recognizing Ki67 (Abcam), and examined with Olympus confocal laser scanning microscope (Olympus).

### Cell culture

Primary hepatocytes of 8-week old mice were isolated using the standard method of *in situ* collagenase (Gibco) perfusion and digestion of liver with low-speed centrifugation (50 g, 1 min), as previously reported^36^,^37^. Isolated cells were plated on collagen type I-coated dishes in Dulbecco's modified Eagle's medium (DMEM) with 10% (v/v) fetal bovine serum (FBS), 100 IU/mL penicillin, and 100 μg/ml streptomycin. Hepatocytes were incubated for 6 h at 37°C in a humidified atmosphere with 95% air and 5% CO_2_, allowing for cell attachment to the plate. The medium was then changed, which involved replacement by 0.1% FBS contained DMEM with or without EGF or HGF for stimulation at indicated times.

### Western blotting analyses

Total protein was isolated from frozen liver tissue and cultured cells with TBS (20 mM Tris, 150 mM NaCl, PH 7.4) containing 1% triton X-100. Protein concentration was measured using a bicinchoninic acid protein assay kit (Thermo Scientific). Equal protein samples were separated by SDS-PAGE and then transferred onto nitrocellulose or polyvinylidinedifluoride (Millipore) membranes. After blocking with 5% skim milk, the membranes were incubated with specific antibodies against the indicated antibodies at 4°C overnight, followed by incubation with horseradish peroxidase-conjugated secondary antibody. Immunoreactivity was visualized by HRP substrate peroxide solution (Millipore). The related antibodies that are used included ERK1 (BD), phospho-ERK, phospho-AKT, AKT, phospho-Met (Tyr1234/5), c-Met, phospho-EGFR (Tyr1068), EGFR, rabbit IgG (Cell Signaling) and mouse IgG (Sigma).

### Enzyme activity assays for Fut8

Frozen liver tissues were homogenized in TBS containing 1% protease inhibitor cocktail (Nacalai tesque Inc.). After centrifugation at 900 g for 10 min, the supernatant was collected for enzyme activity assays. Each sample containing 800 μg of total protein was centrifuged at 105,000 g for 1 h, then the pellet was resuspended in 0.1 M MES-NaOH (PH 7.0) for reactions. Equal amounts of protein were used in Fut8 activity assays. The specific activities of Fut8 were determined using a substrate, 4-(2-pyridylamino)-butyl-amine (PABA)-labeled GlcNAcβ1-2Manα1-6(GlcNAcβ1-2Mana1-3)Manβ1-4GlcNAcβ1-4GlcNAc-Asn (GnGn-Asn-PABA). Each assay used 2 mM of acceptor substrate and 2 mM GDP-L-fucose as a donor (in 10 μl of total reaction solution). The reactions were terminated by boiling after 2 h of incubation at 37°C, and the reaction mixtures were centrifuged at 10,000 g for 10 min. The result supernatants were applied to high-performance liquid chromatography (HPLC) equipped with a TSK-gel, ODS-80TM column (4.6 × 150 mm) in order to separate and quantitate the products. Elution was performed isocratically at 55°C using a 20 mM acetate buffer (pH 4.0) containing 0.15% butanol. The column eluate was monitored for fluorescence using a detector operating at excitation and emission wavelengths of 320 and 400 nm, respectively. The activities of endogenous Fut8 were measured by HPLC, expressed as the pmol of fucose transferred/h/mg of proteins^38^.

### Statistical analysis

Results are given as the mean ± standard error of the mean (SEM). The data were analyzed using Prism 5.0 software (GraphPad Software Inc.). Comparisons were carried out using 2-tailed Mann-Whitney tests and/or a Tukey's Multiple Comparison test. A *P* value of less than 0.05 was considered significant.

## Author Contributions

Y.W. and T.F. performed the all experiments. J.G. designed the project. Y.O., Y.K. and E.M. performed 70% hepatectomy. T.I., G.W. and H.L. analyzed cellular signaling. Y.W., T.F., J.L., N.T. and J.G. analyzed the data. Y.W. and J.G. wrote the manuscript with J.L. All authors discussed the results and commented on the manuscript.

## References

[b1] Taub R. Liver regeneration: from myth to mechanism. Nat. Rev. Mol. Cell Biol. 5, 836–847 (2004). 15459664 10.1038/nrm1489

[b2] Michalopoulos G. K. Liver regeneration. J. Cell Physil. 213, 286–300 (2007). 10.1002/jcp.21172 PMC2701258 17559071

[b3] Michalopoulos G. K. & DeFrances M. Liver regeneration. Adv. Biochem. Eng. Biotechnol. 93, 101–134 (2005). 15791946 10.1007/b99968

[b4] Michalopoulos G. K. Advances in liver regeneration. Expert Rev. Gastroenterol. Hepatol. 8, 897–907 (2014). 24964729 10.1586/17474124.2014.934358

[b5] Si-Tayeb K., Lemaigre F. P. & Duncan S. A. Organogenesis and development of the liver. Dev. Cell 18, 175–189 (2010). 20159590 10.1016/j.devcel.2010.01.011

[b6] Fausto N., Campbell J. S. & Riehle K. J. Liver regeneration. Hepatology 43, S45–53 (2006). 16447274 10.1002/hep.20969

[b7] Boscher C., Dennis J. W. & Nabi I. R. Glycosylation, galectins and cellular signaling. Curr. Opin. Cell Biol. 23, 383–392 (2011). 21616652 10.1016/j.ceb.2011.05.001

[b8] Snider N. T. & Omary M. B. Post-translational modifications of intermediate filament proteins: mechanisms and functions. Nat. Rev. Mol. Cell Biol. 15, 163–177 (2014). 24556839 10.1038/nrm3753 PMC4079540

[b9] Haltiwanger R. S. & Lowe J. B. Role of glycosylation in development. Annu. Rev. Biochem. 73, 491–537 (2004). 15189151 10.1146/annurev.biochem.73.011303.074043

[b10] Wang X. *et al.* Dysregulation of TGF-beta1 receptor activation leads to abnormal lung development and emphysema-like phenotype in core fucose-deficient mice. Proc. Natl. Acad. Sci. U S A 102, 15791–15796 (2005). 16236725 10.1073/pnas.0507375102 PMC1257418

[b11] Wang X. *et al.* Core fucosylation regulates epidermal growth factor receptor-mediated intracellular signaling. J. Biol. Chem. 281, 2572–2577 (2006). 16316986 10.1074/jbc.M510893200

[b12] Zhao Y. *et al.* Deletion of core fucosylation on alpha3beta1 integrin down-regulates its functions. J. Biol. Chem. 281, 38343–38350 (2006). 17043354 10.1074/jbc.M608764200

[b13] Gu W. *et al.* alpha1,6-Fucosylation regulates neurite formation via the activin/phospho-Smad2 pathway in PC12 cells: the implicated dual effects of Fut8 for TGF-beta/activin-mediated signaling. FASEB J. 27, 3947–3958 (2013). 23796784 10.1096/fj.12-225805

[b14] Hutchinson W. L., Du M. Q., Johnson P. J. & Williams R. Fucosyltransferases: differential plasma and tissue alterations in hepatocellular carcinoma and cirrhosis. Hepatology 13, 683–688 (1991). 1849114

[b15] Takahashi T. *et al.* alpha1,6fucosyltransferase is highly and specifically expressed in human ovarian serous adenocarcinomas. Int. J. Cancer 88, 914–919 (2000). 11093814 10.1002/1097-0215(20001215)88:6<914::aid-ijc12>3.0.co;2-1

[b16] Chen C. Y. *et al.* Fucosyltransferase 8 as a functional regulator of nonsmall cell lung cancer. Proc. Natl. Acad. Sci. U S A 110, 630–635 (2013). 23267084 10.1073/pnas.1220425110 PMC3545778

[b17] Muinelo-Romay L. *et al.* Expression and enzyme activity of alpha(1,6)fucosyltransferase in human colorectal cancer. Int. J. Cancer 123, 641–646 (2008). 18491404 10.1002/ijc.23521

[b18] Sano K. *et al.* Survival signals of hepatic stellate cells in liver regeneration are regulated by glycosylation changes in rat vitronectin, especially decreased sialylation. J. Biol. Chem. 285, 17301–17309 (2010). 20335177 10.1074/jbc.M109.077016 PMC2878493

[b19] Yang X., Tang J., Rogler C. E. & Stanley P. Reduced hepatocyte proliferation is the basis of retarded liver tumor progression and liver regeneration in mice lacking N-acetylglucosaminyltransferase III. Cancer Res. 63, 7753–7759 (2003). 14633700

[b20] Becker D. J. & Lowe J. B. Fucose: biosynthesis and biological function in mammals. Glycobiology 13, 41R–53R (2003). 10.1093/glycob/cwg054 12651883

[b21] Hidalgo A. *et al.* Insights into leukocyte adhesion deficiency type 2 from a novel mutation in the GDP-fucose transporter gene. Blood 101, 1705–1712 (2003). 12406889 10.1182/blood-2002-09-2840

[b22] Borowiak M. *et al.* Met provides essential signals for liver regeneration. Proc. Natl. Acad. Sci. U S A 101, 10608–10613 (2004). 15249655 10.1073/pnas.0403412101 PMC490025

[b23] Natarajan A., Wagner B. & Sibilia M. The EGF receptor is required for efficient liver regeneration. Proc. Natl. Acad. Sci. U S A 104, 17081–17086 (2007). 17940036 10.1073/pnas.0704126104 PMC2040457

[b24] Bohm F., Kohler U. A., Speicher T. & Werner S. Regulation of liver regeneration by growth factors and cytokines. EMBO Mol. Med. 2, 294–305 (2010). 20652897 10.1002/emmm.201000085 PMC3377328

[b25] Paranjpe S. *et al.* Cell cycle effects resulting from inhibition of hepatocyte growth factor and its receptor c-Met in regenerating rat livers by RNA interference. Hepatology 45, 1471–1477 (2007). 17427161 10.1002/hep.21570 PMC2632963

[b26] Huh C. G. *et al.* Hepatocyte growth factor/c-met signaling pathway is required for efficient liver regeneration and repair. Proc. Natl. Acad. Sci. U S A 101, 4477–4482 (2004). 15070743 10.1073/pnas.0306068101 PMC384772

[b27] Speicher T. *et al.* Knockdown and knockout of beta1-integrin in hepatocytes impairs liver regeneration through inhibition of growth factor signalling. Nat. Commun. 5, 3862 (2014). 24844558 10.1038/ncomms4862

[b28] Ferrara C. *et al.* Unique carbohydrate-carbohydrate interactions are required for high affinity binding between FcgammaRIII and antibodies lacking core fucose. Proc. Natl. Acad. Sci. U S A 108, 12669–12674 (2011). 21768335 10.1073/pnas.1108455108 PMC3150898

[b29] Mizushima T. *et al.* Structural basis for improved efficacy of therapeutic antibodies on defucosylation of their Fc glycans. Genes Cells 16, 1071–1080 (2011). 22023369 10.1111/j.1365-2443.2011.01552.x PMC3258418

[b30] Okazaki A. *et al.* Fucose depletion from human IgG1 oligosaccharide enhances binding enthalpy and association rate between IgG1 and FcgammaRIIIa. J. Mol. Biol. 336, 1239–1249 (2004). 15037082 10.1016/j.jmb.2004.01.007

[b31] Shinkawa T. *et al.* The absence of fucose but not the presence of galactose or bisecting N-acetylglucosamine of human IgG1 complex-type oligosaccharides shows the critical role of enhancing antibody-dependent cellular cytotoxicity. J. Biol. Chem. 278, 3466–3473 (2003). 12427744 10.1074/jbc.M210665200

[b32] Jimenez-Romero C. *et al.* Using old liver grafts for liver transplantation: Where are the limits? World J. Gastroenterol. 20, 10691–10702 (2014). 25152573 10.3748/wjg.v20.i31.10691 PMC4138450

[b33] Fukuda T. *et al.* Alpha1,6-fucosyltransferase-deficient mice exhibit multiple behavioral abnormalities associated with a schizophrenia-like phenotype: importance of the balance between the dopamine and serotonin systems. J. Biol. Chem. 286, 18434–18443 (2011). 21471224 10.1074/jbc.M110.172536 PMC3099660

[b34] Gao L. *et al.* Reticulon 4B (Nogo-B) facilitates hepatocyte proliferation and liver regeneration in mice. Hepatology 57, 1992–2003 (2013). 23299899 10.1002/hep.26235 PMC3628958

[b35] Mitchell C. & Willenbring H. A reproducible and well-tolerated method for 2/3 partial hepatectomy in mice. Nat. Protoc. 3, 1167–1170 (2008). 18600221 10.1038/nprot.2008.80

[b36] Tokairin T. *et al.* A highly specific isolation of rat sinusoidal endothelial cells by the immunomagnetic bead method using SE-1 monoclonal antibody. J. Hepatol. 36, 725–733 (2002). 12044521 10.1016/s0168-8278(02)00048-x

[b37] Maher J. J., Bissell D. M., Friedman S. L. & Roll F. J. Collagen measured in primary cultures of normal rat hepatocytes derives from lipocytes within the monolayer. J. Clin. Invest. 82, 450–459 (1988). 3042806 10.1172/JCI113618 PMC303534

[b38] Uozumi N. *et al.* Purification and cDNA cloning of porcine brain GDP-L-Fuc:N-acetyl-beta-D-glucosaminide alpha1-->6fucosyltransferase. J. Biol. Chem. 271, 27810–27817 (1996). 8910378 10.1074/jbc.271.44.27810

